# Assessing the validity and reliability of the Malagasy version of Oral Impacts on Daily Performance (OIDP): a cross-sectional study

**DOI:** 10.1186/s13030-016-0087-z

**Published:** 2017-02-02

**Authors:** Noeline Razanamihaja, Eva Ranivoharilanto

**Affiliations:** 1 IOSTM, University of Mahajanga, Mahajanga, Madagascar; 2 University of Paris Diderot, Paris VII, France; 3 Ministry of Public Health, Antananarivo, Madagascar

**Keywords:** Oral Impacts on Daily Performance - OIDP, Malagasy, Prevalence, Reliability, Validity

## Abstract

**Background:**

Evaluating health needs includes measures of the impact of state of health on the quality of life. This entails evaluating the psychosocial aspects of health. To achieve this, several tools for measuring the quality of life related to oral health have been developed. However, it is vital to evaluate the psychometric properties of these tools so they can be used in a new context and on a new population. The purpose of this study was to evaluate the reliability and validity of the Malagasy version of a questionnaire for studying the impacts of oral-dental health on daily activities (Oral Impacts on Daily Performance), and analyse the interrelations between the scores obtained and the oral health indicators.

**Method:**

A cross-sectional study was performed for the transcultural adaptation of the Oral Impacts on Daily Performance questionnaire forward translated and back-translated from English to Malagasy and from Malagasy to English, respectively. The psychometric characteristics of the Malagasy version of the Oral Impacts on Daily Performance were then evaluated in terms of internal reliability, test-retest, and construct, criteria and discriminant validity. Four hundred and six adults responded in face-to-face interviews to the Malagasy version of the Oral Impacts on Daily Performance questionnaire.

**Results:**

Nearly 74% of the participants indicated impacts of their oral health on their performance in their daily lives during the 6 months prior to the survey. The activities most affected were: “smiling”, “eating” and “sleeping and relaxing”. Cronbach’s alpha was 0.87. The construct validity was demonstrated by a significant association between the Oral Impacts on Daily Performance scores and the subjective evaluation of oral health (*p* <0.001). Discriminant validity was demonstrated by the fact that the Oral Impacts on Daily Performance scores were significantly higher in subjects with more than ten missing teeth, compared to those with fewer than ten missing teeth (*p* < 0.001).

**Conclusion:**

The Malagasy version of the Oral Impacts on Daily Performance index is a valid and reliable measure for use in Malagasy adults over 55 years old.

## Background

The measure of health related quality of life has increased considerably over the last few decades, and it is currently accepted as an essential element in investigations in oral health, especially to acquire characteristics of a non-clinical nature and felt by the patients. It entails evaluating the psychosocial aspects of health. Indeed, oral health problems can have impacts on the daily activities of patients. Thus a certain number of tools for measuring the quality of life related to oral health, such as the Oral Health Related Quality of Life (OHRQoL), have been developed [1]. The Oral Impacts on Daily Performance (OIDP) is an international socio-dental indicator of the quality of life related to oral health, commonly used to evaluate the impacts of oral health conditions on the capacities of individuals to perform daily activities [2]. The OIDP is a generic measurement instrument developed by Adulyanon et al. in 1996 [3] and then adapted in several countries (Greece, United Kingdom, Tanzania, Uganda, Norway, France, Iran, Japan, Spain, Korea, Sweden, Bosnia-Herzegovina, India, Chili, Nigeria, Croatia) [4–19] where its psychometric properties were tested and deemed adequate, valid and reliable by cross-sectional studies. OIDP can be used in different age groups of people, in separated or in only one studies. It has been shown to be applicable to elderly people in Tanzania [6] and Bosnia [15], for children and adolescent in France [9], Uganda [7] and Nigeria [18]. A version for children is available, the Child-OIDP (C-OIDP).

Results from rare surveys conducted in Madagascar showed high level of oral diseases within the population, and particularly early tooth loss within adults aged 35 years old and above. Most of time, these oral diseases are left untreated [20]. Total edentelousness prevalence among 65 years old Malagasy people is high compared to that found in some African countries [21].

Like in many countries, Madagascar knew these two past decades the growing ageing populations. In this context and in order to oral health service planning for this age group, the OIDP index is worthy of consideration because of its adaptation for use in oral health needs assessment.

In Madagascar, there is no such questionnaire validated for use with the adult population, which constitute barriers to the development of this kind of research on population. The OIDP has never been adapted for Madagascar.

Prior to using a new measurement scale in a new context and new group of population, it was important to re-evaluate its psychometric properties.

The objectives of this study were to develop and test the reliability and validity of a Malagasy version of the OIDP questionnaire on a sample of Malagasy elderly adults.

## Methods

A descriptive cross-sectional study was performed. According to statistics, in 2012, nearly 35% of adults in Madagascar were illiterate. Therefore the face-to-face interview method was applied in this study for those respondents unable to complete the questionnaire themselves. The OIDP questionnaire included 8 items, distributed in three dimensions: physical (03 items), psychological (03 items) and social (02 items) performance. It evaluated the degree to which the daily life of a person was negatively affected by their state of oral health.

The OIDP posed questions on the difficulties of performing eight daily activities (eating, speaking, tooth-brushing, sleeping, controlling emotions, smiling, working, and appreciating social contacts) related to oral health. The questionnaire was organized in two parts: in the first part, the respondents were asked to list the dental problems that they had to cope with during the previous six months. If the respondent expressed an impact on one of these activities, they had to indicate the severity and frequency of the impact by using scores from 1 to 3 for each one. The “impact” was recorded immediately the subject expressed having had a problem in one of the daily activities mentioned above. If no impact was perceived, the activity was assigned a “zero” score.

### Recruitment of the respondents

The study population was composed of adults aged 55 years old or older. Recruitment was subject to the consent of the subjects to participate in the study.

Were included in the study people aged 55 years and over, with the ability to answer questions directly.

The elderly suffering from language disorder, hearing, mental handicap of depression type or relational difficulties were excluded.

The study was performed in the capital city, Antananarivo and its suburbs.

The validation process consisted of three steps: translation; qualitative validation including all the pre-tests and test-retests; and quantitative validation steps which included different internal and external reliability and validity tests, and construct, criterion and discriminant tests.

### Forward translation and back-translation process

The OIDP questionnaire was first translated from English to Malagasy by two native bilingual persons, one a dentist and the other a linguist. The translators came up against several difficulties in expressing the concept of quality of life related to oral health in the Malagasy context. A committee of experts composed of translators and authors discussed the problematic points of the translation in order to reach consensus. A synthesis of the two translations was established to obtain a single preliminary version in Malagasy. The latter was pre-tested on 20 adult subjects. After each interview, a discussion with the respondents was held to check whether the phrasing, vocabulary and method of administering the questionnaire were pertinent and whether difficulties existed in understanding the items. Following this pre-test, minor modifications were made as certain words required dialectic equivalences. The finalised version was then retranslated into English by two new translators in conformity with the forward translation back-translation method [22, 23] to verify whether the Malagasy version correctly expressed the same content as the items of the original questionnaire. Minimal differences in the back-translation were discussed by a committee of experts. Several modifications were made to the wording of the phrases to match Malagasy conceptions and a penultimate version of the questionnaire was obtained. This version was then tested and retested 15 days afterwards, with about 60 subjects that did not take part in the pre-test or belong to the final study population, in view to evaluating the reproducibility, stability of the responses and the validity of the content.

### Validation of the Malagasy version of the questionnaire

This step involved the participation of 406 adults.

Before administering the questionnaire, a clinical examination was performed by two calibrated dentists and the evaluation of oral health was carried out on the basis of indicators recommended by the World Health Organization (WHO) [24]. The aim was to determine the number of natural teeth in the mouth, prosthesis use and requirements, and to describe the state of periodontal health.

### Data analysis

The data permitted calculating a score for each activity and a global score. The OIDP scores were expressed by the sum of the different performance scores (severity score × frequency score) divided by the maximum possible global score, then multiplied by 100 to obtain a percentage score. The global score could range from 0 to 72; it was then divided by 72 and then multiplied by 100. Thus the final score of the questionnaire varied from 0 to 100. The highest OIDP scores expressed a lower quality of life.

The analysis was performed with the Statistical Package for Social Sciences (SPSS) software, version 18.0 for Windows. The threshold of statistical significance was set at 0.05.

The face validity and content validity were tested during a pre-test.

The reproducibility of the responses to the Malagasy version of the OIDP was evaluated during the *test-retest* by Intra-class Correlation Coefficients (ICC) where the intra-individual and the inter-individual variations divided by the total variation were expressed as a ratio between 0 and 1. It is generally accepted that a coefficient at least equal to 0.7 is required to conclude on acceptable test-retest reproducibility.

### Evaluation of the validity of the questionnaire

The descriptive analysis of the scores of the items was carried out first after which the *external validity* was evaluated by the strength of the correlation between the positive measures. The *internal consistency reliability* of the items was evaluated by calculating Cronbach’s alpha if the items were eliminated. A coefficient larger than 0.70 indicated acceptable reliability [25].

Since the OIDP scores were not normally distributed, Kruskal–Wallis and Mann–Whitney nonparametric tests were used to evaluate the construct validity. As no gold standard was available, it was difficult to establish the criterion validity.

Factor analyses including confirmatory factor analysis was performed based on the three dimensions of OIDP.

It is commonly agreed that tooth loss, in most cases is a consequence of oral diseases, and affects Oral Health-Related Quality of Life (OHRQoL). In this scope, the study was aimed to discriminate groups with reported dental pain in one hand and between subjects having or not more than ten missing and not replaced teeth in other hand.

## Results

Four hundred and six people out of the 430 contacted accepted to participate in the study, i.e. a response rate of 94.5%.

### The characteristics of the study population are presented in Table 1

**Table 1:** characteristics of the study population

Characteristics		Number	Percent
Gender	Male	178	43.8
	Female	228	56.2
Age group	55–60 years old	285	70.3
	61–64 years old	29	13.9
	65–70 years old	13	6.2
	71 years and older	20	9.8
Social status	Living alone	121	30.2
	Lives in couple	254	63.02
Education level	Never been at school	28	6.9
	Unfinished primary school	72	17.8
	Unfinished secondary school	66	16.3
	A finished secondary school	81	20.0
	High school, university	92	22.8
	Do not know	9	0.1
Do you smoke	Yes	89	21.9

The average age was 60.89 years with a standard deviation 7.56. There were slightly more women (56.2%) than men. 63.02% of the respondents lived in couples. Slightly more than half of them had stopped their education at secondary school (51.4%). The prevalence of tobacco consumption was 21.9%.

### Oral health status

The average Decayed, Missing, Filled Teeth (DMFT) was 16.51 with a standard deviation of 8.88 and only 2.8% of the study population had no caries. The index was dominated by the Missing Teeth (MT) component for missing teeth of 11.94 (9.09). Total edentulousness of both mandibles was recorded in 9% of subjects and 46.9% had more than 10 missing teeth. Nearly two thirds required dental prosthetics. More than one third of the respondents said that they were handicapped by dental pain 6 months prior to the survey (Table 2).

**Table 2:** Percentage distribution of clinical status in malagasy eldery subjects (*n* = 209)

Clinical state	Categories	Indices Mean (SD)
Mean teeth number	Tooth decay (DT)	3.58 (3.90)
	Missing teeth (MT)	11.94 (9.09)
	Filled teeth (FT)	0.99 (2.57)
	DMFT	16.51 (8.88)
Denture status	Both edentulous	*N* (%) 30 (8.9)
	Upper edentulous	41 (10.2)
	Lower edentulous	23 (5.7)
Number of natural teeth	0	18 (7.7)
	1–10	106 (50.7)
	11–20	51 (24.4)
	21 and more	36 (17.2)
Prosthetics needs reported	No need	103 (25.4)
	In one jaw	53 (13.2)
	In both jaw	246 (61.2)
Perceived dental pain	No pain	227 (56.3)
	Moderate pain	115 (28.5)
	Acute pain	61 (15.1)
Perceived oral health status	Healthy	162 (40.2)
	Poor oral health	241 (59.8)

### Impacts of the state of oral health on the quality of daily life

Table 3 shows that 6 months prior to the survey, psychological (46.43%) and physical (45.06%) performance were the areas subject to the greatest impacts of the state of oral health. Nearly 74% of the respondents reported at least one impact and the daily activities most affected were “smiling”, “eating” and “sleeping, relaxing”, for 61.8, 59.8 and 40.0%, respectively. All the other activities were affected at levels between 37 and 39%.

**Table 3:** Percentage distribution (percentage of adults affected less than once a month or more) and mean frequency scores (SD) for the eight OIDP items and the OIDP ADD and OIDP SC scores

Affected:	Yes *N* (%)	Mean (SD)
Physical performances (45.06%)		
1. Eating and enjoying food	241 (59.8)	1.93 (2.02)
2. Speaking and pronouncing clearly	152 (37.7)	1.24 (1.85)
3. Cleaning teeth	152 (37.7)	1.07 (1.68)
Psychological performances (46.43%)		
4. Sleeping and relaxing	161 (40.0)	1.00 (1.49)
5. Smiling, laughing	154 (61.8)	1.23 (1.81)
6. Maintaing usual emotional status without being irritable	151 (37.5)	0.92 (1.46)
Social performances (37.8%)		
7. Carrying out major work or social role	157 (38.9)	0.86 (1.32)
8. Enjoying contact with people	148 (36.7)	0.90 (1.41)
OIDP SC total scores	298 (73.9)	3.27 (2.95)
OIDP ADD scores	-	9.14 (9.55)

### Reliability tests of the Malagasy OIDP

During the test retest, the Malagasy OIDP presented an intra-class correlation coefficient of 0.895 with a Confidence Interval (CI) of 95% (0.878–0.910) at *p* <0.001. In this study, we did not find any significant association between the OIDP scores according to (as a function of) gender and age (*p* > 0.05).

The inter-item correlation coefficients of the Malagasy OIDP ranged from *r* = 0.37 (relation between the problem of tooth cleaning and eating) at *r* = 0.67 (relation between smiling and speaking). There was no negative value (Table 4).

**Table 4:** Reliability analysis of OIDP index for Malagasy participants: OIDP items Correlation matrix (1–8)

	1	2	3	4	5	6	7	8
1. Eating	1,00							
2. Speaking	0.484^**^	1,00						
3. Cleaning teeth	0.370^**^	0.470^**^	1,00					
4. Sleeping and relaxing	0.489^**^	0.392^**^	0.456^**^	1				
5. Smiling, laughing	0.504^**^	0.673^**^	0.401^**^	0.398^**^	1			
6. Maintaining usual emotional status	0.484^**^	0.499^**^	0.465^**^	0.629^**^	0.487^**^	1		
7. Carrying out major work	0.466^**^	0.403^**^	0.424^**^	0.582^**^	0.480^**^	0.666^**^	1	
8. Enjoying contact with people	0.399^**^	0.450^**^	0.389^**^	0.397^**^	0.555^**^	0.560^**^	0.542^**^	1

**p <0.001

Table 5 shows the results of the internal reliability test with a Cronbach alpha of 0.87. Eliminating the different items did not lead to any increase in this value.

**Table 5:** Analysis of interne coherence of OIDP items: complete correlation of corrected items, Cronbach’s alpha if items deleted. Reliability analysis: Corrected item-total correlations

Performances	Corrected item-total correlation	Cronbach’s alpha if items deleted
Eating	0.610	0.86
Speaking and pronouncing clearly	0.657	0.85
Cleaning teeth	0.560	0.86
Sleeping and relaxing	0.631	0.86
Smiling, laughthing	0.681	0.85
Carring work	0.724	0.85
Maintaining emotional status	0.676	0.85
Enjoying contact with other people	0.624	0.86
Global Cronbach’alpha for the 8 items : 0.876		

The results of the construct and criterion validation are presented in Table 6. The problems caused by the absence or not of teeth felt by the respondents matched with the mean scores of the OIDP (*p* < 0.001).

**Table 6:** Construct validity and criterion validity of OIDP questionnaire scores in relation with clinical measures on the sample (*n* = 403)

Variables	Categories	*N* (%)	Score OIDP	percentiles	*p*
Prosthetics needs reported			Kruskal Wallis		
			Khi^2^ = 21.96	[0,0-2,0-2,0]	<0.001
	No need	103 (23.6)	27.91	[0,0-0,0-1,0]	<0.001
	on 01 maxillary	53 (13.02)	2.19	[0,0-0,0-0,0]	0.134
	on 02 maxillaries	246 (61.00)	34.02	[0,0-1,0-1,0]	<0.001
Dental pain reported			Mann & Whitney		
			0.59 (0.739)	[0,0-0,0-1,0]	<0.001
	No pain	227 (56.3)	12.18 (14.35)	[0,0-1,0-1,0]	
	Medium dental pain	115 (28.5)	26.15 (17.92)	[0,0-0,0-1,0]	
Perceived difficulty to eat			Mann & Whitney		
			0.60 (0.49)		<0.001
	No difficulty to eat	162 (40.2)			
	Poor oral health	241 (59.8)		[0,0-1,0-1,0]	

The factor analysis assessment results showed that KMO value, 0.698, was acceptable in the present study. The results showed that the first dimension (physical performances) explained 80.32% of variances (Table 7).

**Table 7:** Factor analysis (principal component analysis) with VARIMAX rotation

Dimension of OIDP	Mean	SD	Number
Physical performances	4,24	4,409	403
Psychological performances	3,14	3,894	403
Social performances	1,75	2,402	403
Indice KMO et test de Bartlett			
Kaiser-Meyer-Olkin sampling precision measure.			,698
Test de sphéricité de Bartlett	Khi^2^ approximated		672,428
	ddl		3
	Signification de Bartlett		,000
Factors	Eigenvalues variables explained		
	Total	% variance	% cumulated
1	2.410	80.326	80.326
2	0.394	13.134	93.459
3	0.196	6.541	100.000
	Extraction Sommes des carrés des facteurs retenus		
	Total	% de la variance	% cumulés
1	2.410	80.326	80.326
2			
3			

Méthode d’extraction : Analyse en composantes principals (ACP)

Regarding the criterion validity test, the statistical significance (*p* <0.001) was observed for the comparison of the OIDP scores between the different categories of self-declared state of oral health (perceived pain, difficulty in eating). As for the construct validity, the scores of individuals who perceived needs for prosthetic care were much higher than those who thought they had no need of it (*p* <0.001).

### Discriminant validity

As shown in Table 8 significant differences were found between groups having or not reported dental pain and in Table 9, significant differences were observed between the group with more than 10 missing teeth (MT > 10) and that with MT < 10. The participants with MT > 10 were 4.10 times (2.674–6.299) and 1.85 times (1.217–2.812) more numerous in reporting emotional, irritability and eating difficulties than the MT < 10 group.

**Table 8:** Discriminant validity: percentage of distribution: (%) number and Odds ratio of simple scores of OIDP (SC) according to dental pain

Affected:	No Dental pain *N* (%)	Dental pain *N* (%)	OR (95% IC)	p*
1. Eating and enjoying	84 (37.0)	157 (89.2)	14,067 [8.140–24.310]	0.000
2. Speaking and pronouncing clearly	68 (30.0)	84 (47.7)	2.135 [1.417–3.317]	0.000
3. Cleaning teeth	71 (31.3)	81 (46.0)	1.873 [1.246–2.818]	0.002
4. Sleeping and relaxing	36 (15.9)	125 (71.0)	13.004 [8.026–21.089]	0.000
5. Smiling, laughing	63 (27.8)	91 (51.7)	2.787 [1.841–4.218]	0.000
6. Maintaining usual emotional status without being irritable	43 (18.9)	108 (61.4)	6.796 [4.335–10.656]	0.000
7. Carrying out major work or social role	40 (17.6)	117 (66.5)	9.271 [5.834–14.732]	0.000
8. Enjoying contact with people	51 (22.5)	97 (55.1)	4.237 [2.755–6.517]	0.000

**Table 9:** Discriminant validity: percentage of distribution: (%) number and Odds ratio of simple scores of OIDP (SC) according to number of missing teeth (MT)

Affected:	MT < 10 *N* (%)	MT > 10 *N* (%)	OR (95% IC)	*p*
1. Eating and enjoying	132 (54.1)	109 (68.6)	1.850 (1.217–2.812)	0.003
2. Speaking and pronouncing clearly	66 (27.0)	86 (54.1)	3.177 (2.086–4.840)	0.000
3. Cleaning teeth	73 (29.9)	79 (49.7)	2.313 (1.528–3.501)	0.000
4. Sleeping and relaxing	81 (33.2)	80 (50.3)	2.038 (1.353–3.069)	0.000
5. Smiling, laughing	62 (25.4)	92 (57.9)	4.031 (2.630–6.177)	0.000
6. Maintaining usual emotional status without being irritable	60 (24.6)	91 (57.2)	4.104 (2.674–6.299)	0.000
7. Carrying out major work or social role	70 (44.6)	87 (55.4)	3.004 (1.978–4.561)	0.000
8. Enjoying contact with people	67 (27.5)	81 (50.9)	2.743 (1.804–4.172)	0.000

## Discussion

This is the first study to have evaluated oral health quality of life using a Malagasy version of the OIDP questionnaire. The translation did not come up against major difficulties apart from the cultural adjustment of terms. The comparison between the first translation and the back-translation did not reveal differences in content. The validity of the content was also established by asking the participants whether they understood the questions in the questionnaire. The size of the sample was not calculated but was large enough to validate the questionnaire for which a minimum of between 50 and 100 subjects is recommended by Altman [26] and between 100 and 200 subjects by Stewart A et al. [1].

The prevalence of negative impacts of oral health was high for this study population. Smiling and eating were the activities most affected by negative impacts. The “eating” item was subject to the greatest impact, as reported for adults in the literature [8, 10, 13, 15, 16, 19] while, the high frequency of difficulties in relaxing and sleeping can be explained by the dental pain described by this study population.

In this study, missing teeth were not replaced despite the fact that two third of the study population presented needs in dental prosthetics, and it was found that caries were also left untreated; the D-T component of the DMFT was 3.58.

Dental health services in developing countries are very limited. Palliative care is not given priority due to its high cost and is often limited to emergency treatment such as the extraction of teeth. Periodontal diseases are frequent [27, 28].

Kaiser recommends values of greater than 0,05 as acceptable. In the present study analysis, the KMO value is 0,698. Bartlett’s measure shows a highly appropriate *p* value of 0,000 [29].

As with Ostberg AL et al., 2008, in Swedish, we did not find a significant association between OIDP scores, gender, age group [8], or level of education, contrary to the study by Montero et al., in Spain, where the differences between genders were reported with women being more affected [12]. When evaluating OIDP reliability by the test retest, which focused on the reproducibility of the values of items when the same subjects responded to the questionnaire twice, the coefficient of correlation was acceptable, higher than 0.7 with a CI of 95% not including the value 1 and *p* < 0.001.

All the inter-item correlations were positive, thus demonstrating good internal consistency and good homogeneity of the items. The values were not high enough to suspect redundancy of responses.

With respect to reliability, the Cronbach alpha higher than 0.80 found in this study was the same as that of J. Eric et al. in 2011, and of Lawal et al. in Nigeria in 2013, indicating the good internal consistency of the Malagasy OIDP [15, 18]. Indeed, Nunnaly and Bernstein suggested that an alpha of 0.70 to 0.90 indicates good internal consistency [30] and Terwee et al., proposed values between 0.70 and 0.95 [31].

The alpha value found in this study was even higher than those of previous studies: Dorri M et al. in Iran, α = 0.79; Naito M et al. in Japan, α = 0.77 and Purohit et al. in India, α = 0.70 [10, 11, 16].

In line with our observations many authors have tested the internal consistency of the OIDP with the Cronbach alpha, but reserves have been expressed regarding the pertinence of this coefficient which can vary according to the number of items on a scale [25].

The validity of the construct and criteria was evaluated by studying the relation between the OIDP scores and the subjective measures (self-reported dental pain, perceived need for prosthetic treatment). The method of only taking into account subjective measures without clinical measures was also employed when validating the Greek OIDP [4]. Indeed, some authors have argued that clinical indicators measure the state of health whereas studying quality of life requires indicators that evaluate psychosocial aspects that can only be expressed subjectively. In this study, the relation between OIDP scores and subjective measures was positively significant and in the direction expected. For example, the worse the state of oral health, the higher the mean OIDP scores were. This means that the construct validity of the Malagasy OIDP was good. Similarly to all the previous validations carried out in other countries, the results of this study showed that the internal reliability test was successful as nearly all the values of the inter-item correlations were above the recommended minimum of 0.20 [25]. The results confirmed the excellent quality of the OIDP for evaluating the impacts of state of oral health on the performance of daily activities.

The Malagasy version of the OIDP for adults was capable of distinguishing the impacts of reported dental pain or not between groups and poor dentition between the group with more than 10 missing teeth and that with fewer missing teeth, and the differences were significantly significant. A higher OIDP score was linked to having 10 or more missing teeth. The same results were obtained in a study carried out on elderly persons in Sweden [14].

The subjects that complained most of positive impacts effectively corresponded to those identified by the clinical examination as having the most absent teeth and this in accordance with the systematic review and meta analysis conducted by Gerritsen et al., which found that subjects with fewer than 10 teeth were twice as likely to report an impact compared with subjects having 21–32 teeth; subject with 11–20 teeth were 1.5 times more likely to report an impact. Moreover, the review indicates that not only number, but also location and distribution of missing teeth affect the severity of OHQoL impairment [32].

Moreover, in this study the F-T component of DMFT showed a nearly null value. The study population had also reported dental pain on the remaining teeth and this is a major problem for these still in activities adults aged 55 to 60 year old which represented two third of the study people.

The limitations of this study concern in particular the problem of seeing the respondents again after they had benefited from treatments to evaluate the reactivity of the questionnaire, by comparing the self-evaluation of the impacts of state of oral health before and after treatment.

## Conclusion

The Malagasy version of the OIDP presented satisfactory psychometric properties. It appeared reliable and valid for use on adult and elderly persons in Madagascar. This enables the use of this instrument in future studies to assess the impact of oral health on Malagasy adults’ quality of life.

## Abbreviations

CI: Confidence interval
DMFT: Decayed, missed, filled teeth
DT: Decayed teeth
FT: Filled teeth
ICC: Intra-class correlation coefficients
MT: Missing teeth
OHRQoL: Oral health related quality of life
OIDP: Oral impacts on daily performance
SPSS: Statistical package for social sciences
WHO: World Health Organization
